# Leveraging Point‐Of‐Care Ultrasound to Diagnose a Rare Arterial Occlusive Thrombus in a Child: A Case Study

**DOI:** 10.1002/jcu.24102

**Published:** 2025-06-02

**Authors:** Vrushali C. Ponde, Neha Singh, Ashok N. Johari, Hanna Smeds, Karen Boretsky

**Affiliations:** ^1^ Anesthesia, Surya Children Hospital Mumbai India; ^2^ Department of Anesthesiology and Critical Care All India Institute of Medical Sciences Bhubaneswar India; ^3^ Surya Children Hospital Mumbai India; ^4^ Department of Physiology & Pharmacology Karolinska Institutet Stockholm Sweden; ^5^ Anesthesiology, Harvard Medical School Boston Massachusetts USA

**Keywords:** arterial thrombus, lower limb ischemia, POCUS, postoperative complication, regional anesthesia, ultrasound

## Abstract

Pediatric arterial thromboembolism is an extremely rare and serious complication, especially rare when it is noncatheter‐related. Most of the literature describes venous and catheter‐related thromboembolism. We report a case of a 10‐year‐old boy with hereditary multiple exostosis who developed acute limb ischemia following deformity correction and Ilizarov ring fixation for limb lengthening. This case highlights two issues: the role of Point of Care Ultrasound (POCUS) and the management of an epidural catheter amidst unanticipated anticoagulation. It also demonstrates the importance of a multidisciplinary team‐based approach for optimum management at each level.

## Graphical abstract

This case report illustrates the successful use of ultrasound imaging of arterial thrombosis in a child following orthopedic surgery, where regular diagnosis was difficult due to the presence of metallic orthopedic frames.

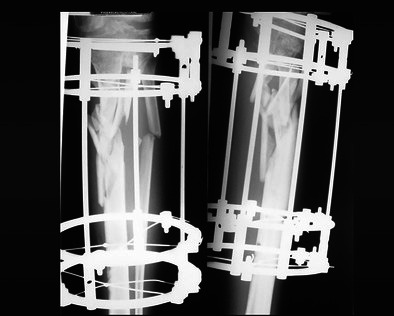

**Abbreviations**
APTT: Activated Prothrombin Time
LMWH: Low Molecular Weight Heparin
MIS‐C: Multisystem Inflammatory Syndrome in Children
PICU: Pediatric Intensive Care Unit
POCUS: Point Of Care Ultrasound
POD: Post Operative Day
US: Ultrasound

## 1 Introduction

Point Of Care Ultrasound (POCUS) is used in various clinical scenarios to provide clear, binary answers, such as ‘yes’ or ‘no’, to clinical questions (Conlon et al. 2019). We present the case of a child undergoing operative limb lengthening who developed limb ischemia on Post Operative Day (POD) 1. Occlusive arterial thrombus is extremely rare in children but should be considered as part of the differential diagnosis (Tuckuviene et al. 2011; Price and Chan 2008; McCrory et al. 2011). POCUS was instrumental in diagnosing femoral artery thrombosis. The ischemic pain was not masked by regional anesthesia. The parents approved the presentation of this case report; no approval from the local ethics committee was required.

### 1.1 Case Report

A 10‐year‐old, 20 kg boy with Ollier's disease and diagnosed with a monomelic‐enchondroma was scheduled for operative placement of an Ilizarov external fixator for limb lengthening with femoral and tibial osteotomies. He had a history of COVID‐19 infection two months prior. His history, examination, and investigations were otherwise unremarkable.

**FIGURE 1:**
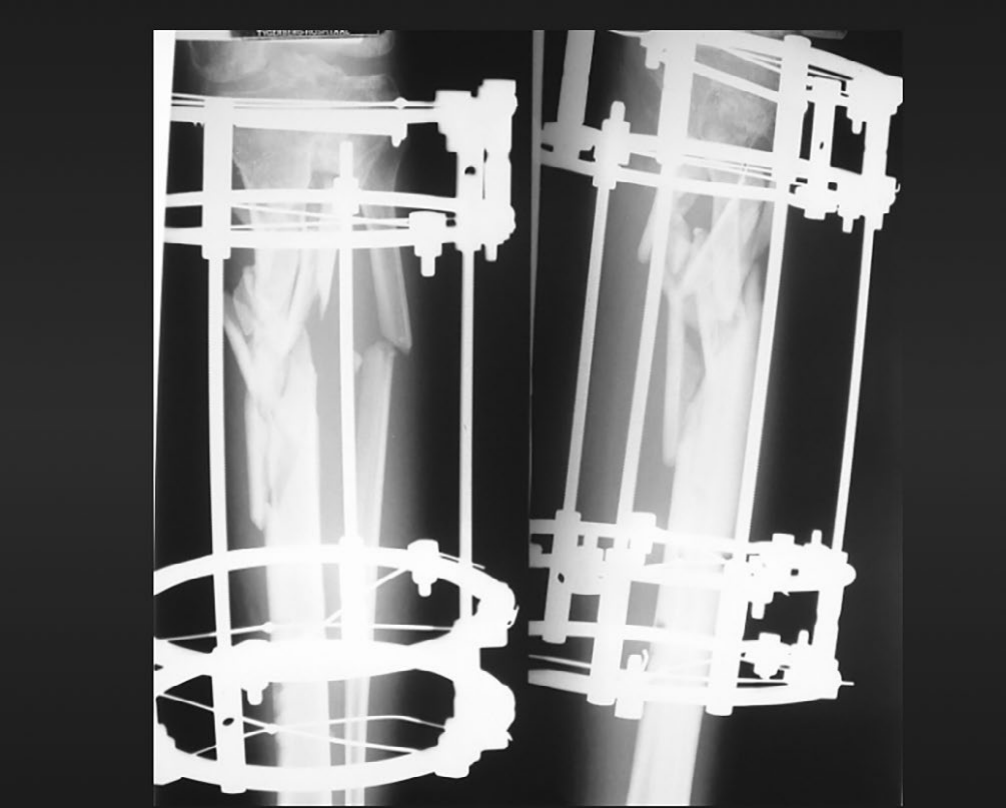
Postoperative x‐ray.

General anesthesia was induced, the airway secured with an endotracheal tube, and a continuous lumbar epidural catheter was placed at the L3‐L4 level under aseptic precautions using an18‐gauge Touhy needle with a 21‐gauge multihole catheter (Portex, Smith medical). A combination of 6 mL of 0.25% bupivacaine with 2 mL of 2% lignocaine and adrenaline was injected into the epidural catheter prior to the surgical incision. Vital signs remained stable throughout the case, indicating successful epidural function. The surgery lasted for 8 h. Epidural analgesia was continued with 0.2% ropivacaine at 3 mL/h through an elastomeric pump. In the immediate postoperative period, the Visual Analog Scale (VAS) pain score was 3 or lower. A postoperative X‐ray is shown in Figure 1.

On POD 1, the patient's foot appeared pale and clammy, the dorsalis pedis pulse was not palpable, and capillary refill time was prolonged. The popliteal pulse was weak. The VAS pain score increased to 4 despite the continued epidural infusion of local anesthetics. The foot ring used for ankle equinus correction was loosened, resulting in partial relief. The epidural infusion was paused to facilitate a potential diagnosis of compartment syndrome. However, the pain intensity escalated rapidly, and the epidural infusion was reinitiated. A bedside ultrasound performed by the anesthesiologist revealed absent pulsations in the popliteal and dorsalis pedis arteries. The findings were compared with the contralateral lower extremity to confirm any differences. A superficial venous thrombosis was observed and documented.

CT angiography showed decreased flow in the right lower limb and increased collateral circulation but could not clearly demonstrate the location and extent of the blockage due to metal artifacts. Therefore, digital subtraction angiography was performed, and a thrombus was located in the superficial femoral artery. The patient underwent emergency embolectomy of the superficial femoral artery under general anesthesia with continued epidural infusion.

Immediately following the embolectomy, portable bedside ultrasound imaging showed return of flow in the popliteal, dorsalis pedis, and posterior tibial arteries. The child was admitted to the Pediatric Intensive Care Unit (PICU). Epidural analgesia continued, and intravenous heparin was administered for 24 h with Activated Prothrombin Time (APTT) monitoring every 8 h. The patient was later transitioned to subcutaneous low molecular weight heparin (LMWH) with APTT monitoring every 24 h. The epidural catheter was removed 24 h after the last dose of LMWH. Recovery was uneventful, although residual foot drop and hypoesthesia of the toes were present at discharge.

## 2 Discussion

This case illustrates the utility of point‐of‐care ultrasound imaging in recognizing pathology and facilitating timely diagnosis and treatment.

Vascular complications following Ilizarov ring application on the femur are rarely reported. Most reported cases involve iatrogenic femoral arterial injury, which typically presents as a delayed pseudo‐aneurysm, a bleeding pin tract often requiring transfusion (Chaikof et al. 1992), or an expansile thigh swelling. Arterial thrombosis following an Ilizarov procedure has not been previously reported, making the diagnosis more challenging. In this case, POCUS facilitated an early diagnosis.

The patient had no typical risk factors (e.g., insertion of transfixation wires in the proximal or mid‐femur) other than a post‐COVID status. Thrombotic complications due to COVID‐19 are well documented, with all three components of Virchow's triad potentially involved: direct endothelial damage by the virus, inflammatory response with cytokine storm, and activation of the coagulation system (Chowdhury et al. 2020; Dain et al. 2022; Tran et al. 2021). While thrombotic events in adults with COVID‐19 are well known, there is limited data on COVID‐19‐associated thrombosis in children. The increased risk in children appears to be associated with older age (> 12 years), cancer, intravascular catheters, critical illness, and MIS‐C (multisystem inflammatory syndrome in children). Most reports conclude that thromboembolic events in children with COVID‐19 are rare (Zaffanello et al. 2021). One retrospective multicenter study reported a 0.7% incidence of thromboembolic events in hospitalized children with asymptomatic SARS‐CoV‐2 (Whitworth et al. 2021).

Lumbar epidural anesthesia for surgical revascularization of the lower extremities has been associated with lower rates of postoperative thrombosis and subsequent interventions compared to general anesthesia alone (Tuman et al. 1991). Regional anesthesia also provides a favorable sympathectomy, improving localized blood flow and aiding in the resolution of ischemia (Aguirre et al. 2012).

This clinical timeline aligns with existing literature indicating that ischemic pain is not masked by regional anesthesia. The key to diagnosis is escalating pain despite effective regional anesthesia. When regional anesthesia is used in patients at risk for acute ischemia, clinicians must remain vigilant for signs of compartment syndrome, including escalating pain, increased swelling, absent sensation, and increased pressure detected by invasive compartment pressure monitoring.

The management of an indwelling epidural in a child receiving unanticipated anticoagulation was guided by APTT values, coagulation profile, and guidelines from the American Society of Regional Anesthesia.

In summary, we present a case of a child with a lower extremity arterial thrombus, a rare complication whose diagnosis was expedited by point‐of‐care ultrasound imaging. We also report that the diagnosis of ischemia was not hindered by the presence of regional anesthesia.

## Author Contributions

V.C.P. helped in conceptualizing and manuscript writing. N.S. helped in manuscript writing and literature search. A.N.J. helped in manuscript writing. H.S. helped in revising the manuscript and handled the submission. K.B. helped in manuscript writing.

## Consent

The parents approved the presentation of this case report on the condition that the patient is anonymized.

## Conflicts of Interest

The authors declare no conflicts of interest.

## Data Availability

The data that support the findings of this study are available on request from the corresponding author. The data are not publicly available due to privacy or ethical restrictions.
